# Exploring the Role of Relationship Dynamics and Chronic Illness in Psychological Outcomes Among Cohabiting Couples During the COVID-19 Pandemic

**DOI:** 10.62641/aep.v53i5.1945

**Published:** 2025-10-05

**Authors:** Laura Lacomba-Trejo, Alda Portugal, Ana Diniz-Vieira, Ashley K. Randall, Ana Paula Relvas

**Affiliations:** ^1^Department of Developmental and Educational Psychology, Faculty of Psychology and Speech Therapy, Universitat de València, 46010 València, Spain; ^2^Department of Psychology, University of Madeira, 9000-072 Madeira, Portugal; ^3^Center for Social Studies, Faculty of Psychology and Education Sciences, University of Coimbra, 3000-115 Coimbra, Portugal; ^4^Faculty of Psychology and Education Sciences, University of Coimbra, 3000-115 Coimbra, Portugal; ^5^Counseling and Counseling Psychology, Arizona State University, Tempe, AZ 85281, USA

**Keywords:** COVID-19, mental health, chronic disease dyadic coping, relationship quality, anxiety

## Abstract

**Background:**

The Coronavirus Disease 2019 (COVID-19) pandemic has exacerbated mental health difficulties among couples. Factors such as chronic physical illness, perceived threat of COVID-19, dyadic coping, and relationship quality may influence levels of psychological distress, including symptoms of anxiety, depression, and stress. This study aimed to examine how these individual and relational variables are associated with psychological outcomes in cohabiting couples during the first national lockdown in Portugal.

**Methods:**

A mixed-methods study was conducted with a sample of 956 individuals (83.9% women), aged 18 to 81 years (*M* = 40.76, *SD* = 10.42), living with a romantic partner for at least one year. Participants completed validated self-report instruments: Depression, Anxiety, and Stress Scales - 21 Items (DASS-21) to assess anxiety, depression, and stress; the Brief Illness Perception Questionnaire (B-IPQ) to evaluate COVID-19 threat perception; the Perceived Relationship Quality Component – Short Version (PRQC-SV) to assess relationship quality; and the Dyadic Coping Inventory (DCI) to measure dyadic coping. Hierarchical linear regression and fuzzy-set Qualitative Comparative Analysis (fsQCA) were used to examine associations between variables. The study received prior approval from the Ethics Committee of the Faculty of Psychology and Educational Sciences of the University of Coimbra.

**Results:**

The regression models accounted from between 17% to 21% of the variances of the dependent variables. In the case of the Qualitative Comparative Analysis (QCA) models, the models explained between 11% and 85% of the cases. Hierarchical regression models (HRMs) showed that COVID-19 threat perception and relationship quality were significantly associated with mental health outcomes. In QCA models, low threat perception and high relationship quality and coping skills correlated with lower psychological distress.

**Conclusions:**

Chronic illness was not significantly associated with psychological distress when compared to COVID-19 threat perception, relationship quality, and dyadic coping. These insights are vital for managing mental health of couples during crises. By underscoring the importance of threat perception, relationship quality, and coping for psychological well-being management during health crises, this study offers valuable insights for supporting couples through periods of adversity.

## Introduction

In March 2020, the World Health Organization (WHO) [1] declared Coronavirus Disease 2019 (COVID-19) a global pandemic, which resulted in nearly seven million deaths worldwide (6,944,971) from its outbreak to 2022 [2]. In Portugal, the first state of emergency was declared on March 18, 2020 [3]. Although it was suspended during certain periods, it only officially ended on April 30, 2021. At this point, some protective measures, such as mandatory masks use in healthcare and nursing home settings, continued to be enforced. By the end of 2023, a total of 27,686 COVID-19-related deaths had been reported in Portugal [2].

During this period, the population experienced significant restrictions on freedom and mobility (e.g., confinement, school closures, curfews), which had substantial impacts not only in terms of the economy, but more critically on mental and physical health. These included interrupted follow-up medical appointments, postponed surgeries, and other disruptions to healthcare access. Some authors have shown that pandemic-related factors—such as separation from one’s family or social network, being deprived of freedom, and feelings of helplessness and uncertainty regarding disease progression—were among the key contributors to widespread emotional and psychological distress in the general population [4, 5, 6].

Within the family context, when couples face stressful situations—whether acute or chronic—such stress impacts both partners individually and as a unit. One of the key consequences of the COVID-19 pandemic was the extent to which it posed a unique challenge to individuals in intimate relationships, underscoring the critical role that these relationships play in psychological and emotional well-being. Previous studies have suggested that the experience of external and chronic stressors has the most significant and negative impact on perceived relationship quality [7, 8, 9, 10]. The pandemic thus emerged as a major stressor for couples, compromising protective factors such as relationship quality and exacerbating risk factors such as gender-based violence [11, 12]. Nevertheless, intimate relationships can also serve as a significant source of emotional support, especially during crises marked by social isolation and major lifestyle disruptions.

Research indicates that overall mental health has deteriorated in comparison to pre-COVID-19 trends, with a general increase in psychological distress (e.g., emotional suffering), marked by symptoms of depression and anxiety—although this pattern does not reflect a continuation of pre-existing upward trends [13]. The highest increases in distress were observed among women, young adults, individuals with prior physical or mental health conditions, and parents of preschool-aged children [13]. Findings regarding physical illness remain inconsistent: one study conducted with a Spanish sample showed that prior mental illness and perceived threat of COVID-19 were strong predictors of both high and low levels of isolation-related distress, whereas the presence of a physical illness was not a determining factor [14, 15].

In relation to the impact of COVID-19 on couples, a cross-cultural study conducted across 27 countries revealed that most participants experienced heightened psychological distress following the implementation of pandemic-related restrictions. This distress was also associated with a lower perceived relationship quality with one’s partner [16]. However, positive dyadic coping has been shown to buffer the psychological distress caused by the various stages of the pandemic and mitigate its negative impacts on both the couple and the individuals [16]. These findings are consistent with prior research (e.g., [17, 18]), which suggests that partner satisfaction and positive dyadic coping can act as a protective factors during crisis such as those triggered by the COVID-19 pandemic.

To the best of our knowledge, few studies have been conducted in Portugal focusing on individuals living with a romantic partner during the COVID-19 lockdown that examine factors associated with anxiety, depression, and stress. Moreover, even fewer have simultaneously covered both linear and non-linear methodologies, as most existing research relies solely on linear models. The integration of these two associative methodologies allow for a more in-depth analysis of the relationship between the variables under study [14]. Fuzzy-set Qualitative Comparative Analyses (fsQCA) enables the identification of multiple combinations or pathways (i.e., equifinality) that can lead to the same outcome.

Therefore, the present study combines linear and non-linear methodologies to identify the factors associated with psychological distress among individuals living with a partner during COVID-19 confinement in Portugal. Specifically, it examines how relationship dynamics interact with individual variables such as chronic physical illness and perceived COVID-19 threat. The main objective of this study is to examine key factors associated with symptoms of anxiety, depression, and stress. We hypothesize that the presence of a chronic physical illness, higher perceived threat of COVID-19, lower relationship quality, and lower levels of positive dyadic coping will be related to higher levels of psychological distress among individuals living in romantic relationships in Portugal.

## Methods

### Procedures

Participants were recruited through institutional websites (e.g., the official webpage of the University of Coimbra) and social media platforms (e.g., Facebook). Informed electronic consent was obtained from all participants before the questionnaires completion. Data were collected online via the Qualtrics platform during the first COVID-19 lockdown in Portugal (April–May 2020). Participation took approximately 25 minutes. The study was conducted in accordance with the Declaration of Helsinki and received ethical approval from the Ethics Committee of the Faculty of Psychology and Educational Sciences, University of Coimbra, on April 3, 2020. At the time of approval, no specific protocol number had been assigned.

### Participants

The final sample consisted of 956 individuals (83.9% women), aged between 18 and 81 years (*M* = 40.76; *SD* = 10.42). Inclusion criteria were: being at least 18 years old, living in Portugal, and being part of a couple living together (cohabitating), whether married or unmarried, for at least one year.

The initial sample comprised 1208 participants. A total of 252 participants individuals were excluded, based on the following criteria: (a) 26 due to the presence of mental health or neurological conditions, (b) 6 who reported both a mental health and a physical health condition, and (c) 220 who reported two or more chronic psychical illnesses. These exclusions were applied to control for the potential cumulative stress factor associated with multiple chronic conditions.

A case-control design was used to explore the impact of chronic physical illness on mental health outcomes. Of the final sample, 20.80% of participants were individuals diagnosed with at least one chronic physical illness and were classified as cases.

The remaining participants, who had no diagnosis of chronic illness (defined as any diagnosed physical or psychological condition requiring ongoing medical follow-up for more than six months), served as the control group. To strengthen internal validity and reduce selection bias, we followed established methodological guidelines for case-control studies, applying a 1:3 matching ratio. Each case was randomly matched with three controls based on age, sex, and other relevant sociodemographic variables [19].

### Data Collection

The variables analyzed were as follows:

(a) Sociodemographic and Medical Variables

An *ad hoc* questionnaire was developed with questions concerning the following information: age, gender and physical or psychological chronic illnesses.

(b) Psychological and Relationship Variables

*Depression, Anxiety, and Stress Scales - 21 Items* (DASS-21): This scale was used to assess symptoms associated with anxiety, depression, and stress in adults and young adults [20] (Portuguese version: [21]). It consists of three subscales (Depression, Anxiety, Stress), each comprising seven items rated on a 4-point Likert scale (0–4), for a total of 21 items. Total scores for each dimension are calculated by summing the respective items [21]. The scale showed adequate psychometric properties [21, 22]. In the present sample, high internal consistency was observed: Depression (Cronbach’s α = 0.92), Anxiety (Cronbach’s α = 0.88), and Stress (Cronbach’s α = 0.92).

*Brief Illness Perception Questionnaire* (B-IPQ): This questionnaire assesses cognitive and emotional representations of the disease [23, 24]. In this study, it was adapted to assess perceived threat related to COVID-19 (e.g., “*To what extent do you feel in control of the COVID-19 situation?*”). This version has seven items since Item 5, which concerns symptom experience, was excluded due to its limited applicability to all participants (e.g., “*How much do you experience symptoms from your illness?*”). While B-IPQ has shown good psychometric properties in prior research [25], the internal consistency in our sample was relatively low (Cronbach’s α = 0.50).

*Perceived Relationship Quality Component – Short Version *(PRQC-SV): Relationship quality was assessed using the short version of the PRQC ([26, 27]; Portuguese version: [28]). This scale includes six items ranging from 1 (“not at all”) to 7 (“extremely”). It has demonstrated psychometric properties in previous studies [26]. In the current sample internal consistency was high (Cronbach’s α = 0.93).

*Dyadic Coping Inventory *(DCI): This instrument assesses the partner’s perception of dyadic coping ([29]; Portuguese version: [30]). It includes 29 items rated on a 5-point Likert scale (1 = “very rarely” to 5 = “very often”). After reversing the required items, all the items were summed to obtain the positive dyadic coping score. The instrument has shown adequate psychometric properties [29] and internal consistency in our sample was excellent (Cronbach’s α = 0.96).

### Data Analysis

Descriptive statistics were performed for all variables under study, and linear regression models were carried out using Statistic Program for Social Sciences (SPSS) v.27 (IBM Corp. Armonk, NY, USA). In parallel, fuzzy-set analysis (fsQCA: 3.2 Charles Ragin and Sean Davey, Irvine, CA, USA) was conducted, eliminating missing data and calculating all the conditions or variables. For dichotomous variables, recalibration involved assigning a value of 0 to indicate absence and 1 to indicate presence of the characteristic. For continuous variables, automatic recalibration was performed using the fsQCA software, based on three qualitative thresholds: 0% (low level or totally out of the set), 50% (intermediate level, neither in nor out of the set) and 90% (high level or totally in the set) [31]. For calibration of continuous variables, we used the fsQCA software’s automatic calibration based on three qualitative anchors: full membership (90th percentile), the crossover point (50th percentile), and full non-membership (10th percentile), following the recommendations of Ragin (2008) [32]. To calculate the total scores, the scales had to be recalibrated. After this, the items were multiplied to obtain the total score [32]. Descriptive values for these conditions were obtained using SPSS v.27.

The fsQCA analysis establishes necessary conditions, ones which must always be present for a certain outcome to occur. Sufficient conditions were also defined, that is, those which can lead to a particular outcome although they do not always have to be present for the outcome to occur. QCA models allow for the identification of the percentage of variance explained, or the cases in which the model is satisfied, (e.g., coverage and goodness-of-fit indicators), consistency [32, 33]. A condition is considered necessary when its consistency is ≥0.90 [32] whereas for sufficiency a model is informative when consistency is around or above 0.74 [33]. In line with fsQCA standards, a solution is considered informative when consistency exceeds 0.74 and coverage values of 0.25 or higher are typically considered meaningful [32].

## Results

### Hierarchical Regression Models vs QCA

#### HRMS

We analyzed the associations between the variables included in this study using a hierarchical regression model (HRM), in which anxiety, depression, and stress were the criterion variables. The explanatory variables included the presence of a chronic physical illness, COVID-19 threat perception, positive dyadic coping, and relationship quality. We established three distinct steps in the model (Table 1): first, the presence of chronic physical illness was included, then the variables considered to be mediators (the perceived threat from COVID-19 and positive dyadic coping) were introduced, and finally, the relationship quality. In all three cases (anxiety, depression, and stress, e.g., emotional distress), in the last step, COVID-19 threat perception and relationship quality showed significant beta coefficients. In fact, these variables explained 17% of the variance in anxiety, 19% of the variance in depression, and 21% of the variance in stress.

**Table 1.** **Hierarchical regression model**.

Independent Variables	Anxiety				Depression				Stress			
	*ΔR* ^2^	*ΔF*	β	*t*	Δ*R*^2^	*ΔF*	β	*t*	Δ*R*^2^	*ΔF*	β	*t*
Step 1	0.00	1.98			0.00	0.60			0.00	0.04		
Chronic physical illness			–0.42	1.22			–0.01	0.31			0.01	0.30
Step 2	0.16	65.53***			0.17	71.09***			0.19	84.09***		
COVID-19 threat perception			0.37	10.87***			0.32	9.29***			0.37	10.98***
Positive dyadic coping			0.02	0.34			–0.07	1.57			–0.06	1.34
Step 3	0.01	9.37**			0.03	22.70***			0.02	17.99**		
Relationship quality			–0.14	3.06**			–0.22	4.77***			–0.19	4.24***
*Durbin-Watson*	1.97				1.92				1.99			
*R^2^_a⁢j⁢d_*	0.17**				0.19***				0.21***			

*Note:* COVID-19, Coronavirus disease 2019; Δ*R,*^2^ Change on *R*^2^; Δ*,* Change on *F*; β, regression coefficient; *t*, t value; **p < 0.01; ***p < 0.001.

### fsQCA

#### Analysis of Necessary Conditions

First, the descriptive and calibration analyses of the fsQCA are presented (Table 2). Secondly, no necessary conditions were identified for the occurrence of high or low levels of anxiety, depression, or stress symptoms, as all consistency values were below 0.90 (Table 3).

**Table 2.** **Descriptive and calibration values of anxiety, depression and stress (DASS-21)**.

	DASS-21			BIPQ	DCI	PRQC
	Anxiety	Depression	Stress	COVID-19 threat perception	Positive Dyadic Coping	Relationship Quality
*M*	122.41	298.10	591.26	538,462.37	3.66	61,720.73
*SD*	921.18	1698.10	1836.43	6,722,772.93	0.85	40,272.87
*Min.*	1.00	1.00	1.00	432.00	1.00	1.00
*Max.*	16,384.00	16,384.00	16,384.00	8,385,300.00	5.00	117,649.00
*P10*	1.00	1.00	1.00	46,656.00	2.57	5048.00
*P50*	2.00	4.00	36.00	311,040.00	3.86	61,740.00
*P90*	96.00	288.00	1458.00	1,296,000.00	4.71	117,649.00

*Note*: *M*, Mean; *SD*, Standard Deviation; *Min*, Minimun; *Max*, Maximum; *P10*, 10th percentile; *P50*, 50th percentile; *P90*, 90th percentile; DASS-21, Depression, Anxiety, and Stress Scales - 21 Items; BIPQ, Brief Illness Perception Questionnaire; DCI, Dyadic Coping Inventory; PRQC, Personal Relationship Quality Component; COVID-19, Coronavirus Disease 2019.

**Table 3.** **Necessary analysis for anxiety, depression and stress (DASS-21)**.

	High levels of anxiety		Low levels of anxiety		High levels of depression		Low levels of depression		High levels of stress		Low levels of stress	
	Cons.	Cov.	Cons.	Cov.	Cons.	Cov.	Cons.	Cov.	Cons.	Cov.	Cons.	Cov.
Presence of chronic physical illness	0.24	0.44	0.21	0.56	0.23	0.38	0.22	0.62	0.23	0.41	0.22	0.60
Absence of chronic physical illness	0.76	0.40	0.79	0.60	0.77	0.37	0.78	0.63	0.77	0.39	0.78	0.61
High levels of COVID-19 threat perception	0.77	0.62	0.56	0.66	0.76	0.56	0.56	0.69	0.78	0.61	0.56	0.66
Low levels of COVID-19 threat perception	0.57	0.48	0.67	0.81	0.58	0.43	0.64	0.82	0.57	0.46	0.67	0.82
High levels of positive dyadic coping	0.60	0.51	0.60	0.74	0.58	0.46	0.61	0.79	0.57	0.47	0.61	0.76
Low levels of positive dyadic coping	0.69	0.54	0.61	0.69	0.73	0.52	0.58	0.70	0.72	0.54	0.58	0.68
High levels of relationship quality	0.58	0.48	0.62	0.74	0.55	0.41	0.63	0.80	0.55	0.44	0.64	0.77
Low levels of relationship quality	0.68	0.56	0.56	0.66	0.73	0.53	0.54	0.67	0.73	0.57	0.54	0.65

*Note*: Cons., consistency; Cov., coverage; DASS-21, Depression, Anxiety, and Stress Scales - 21 Items; COVID-19, Coronavirus Disease 2019; Necessary condition: consistency ≥0.90.

#### Analysis of Sufficiency Conditions

All the models performed, except for the model for high levels of depression, were informative. The models are presented in Table 4. Hereafter, where there are more than three explanatory paths, only the three main ones are presented.

**Table 4.** **Sufficiency analysis for high and low levels of stress**.

Frequency cut-off point 1	Highs levels of anxiety		Lows levels of anxiety			Lows levels of depression			Highs levels of stress			Lows levels of stress		
	Consistency cut-off point 0.76		Consistency cut-off point 0.85			Consistency cut-off point 0.92			Consistency cut-off point 0.74			Consistency cut-off point 0.85		
	1	2	1	2	3	1	2	3	1	2	3	1	2	3
Chronic physical illness	●	●	○		○			○			●			○
COVID-19 threat perception	●	●	○	○		○	○		●	●		○	○	
Positive dyadic coping	○	●			○		●	○		○	●		●	○
Relationship quality	●	○		●	●	●		●	○		○	●		●
Raw coverage	0.09	0.08	0.52	0.47	0.46	0.45	0.44	0.90	0.59	0.59	0.09	0.47	0.46	0.26
Unique coverage	0.03	0.03	0.11	0.02	0.01	0.03	0.06	0.89	0.06	0.08	0.01	0.03	0.06	0.05
Consistency	0.76	0.77	0.82	0.87	0.86	0.90	0.85	0.87	0.75	0.74	0.74	0.89	0.88	0.86
Overall consistency		0.74			0.81			0.58			0.74			0.60
Overall coverage		0.11			0.69			0.85			0.68			0.84

*Note*: Expected vector according to Fiss nomenclature (2010). For high levels of anxiety, depression and stress: 1-0, 1, 0, 0; For low levels of anxiety, depression and stress: 1-0, 0, 1,1; ●, presence of condition; ○, absence of condition; COVID-19, Corinavirus Disease 2019.

Regarding high levels of anxiety, two sufficient configurations were identified, which together explained 11% of the cases. The most explanatory pathway included the presence of a chronic physical illness and high COVID-19 threat perception, combined with high relationship quality and low positive dyadic coping. The second configuration also involved chronic physical illness and high COVID-19 threat perception but combined with high positive dyadic coping and low relationship quality.

As for low levels of anxiety, three configurations explained 69% of the cases. The most explanatory pathway combined the absence of both chronic physical illness and COVID-19 threat perception. The second involved low threat perception and high relationship quality. The third pathway consisted of the absence of chronic illness and low dyadic coping, along with high relationship quality.

In the case of low depression levels, three configurations accounted for 85% of the cases. The most explanatory combination included low COVID-19 threat perception and high relationship quality. The second pathway combined low threat perception with high positive dyadic coping. The third resulted from the absence of chronic illness and the presence of both positive dyadic coping and high relationship quality.

Regarding high levels of stress, three sufficient pathways were found, explaining 68% of the cases. The most explanatory one involved high COVID-19 threat perception and low relationship quality. The second consisted of high threat perception and low positive dyadic coping. The third involved chronic illness, high dyadic coping, and low relationship quality.

Finally, three configurations explained 84% of the cases of low stress levels. The most explanatory pathway combined low COVID-19 threat perception with high relationship quality. The second involved low threat perception and high dyadic coping. The third consisted of the absence of chronic illness and high dyadic coping, along with high relationship quality.

## Discussion

Few studies have evaluated the impact of COVID-19 on individuals living with a partner in Portugal during the confinement imposed by the pandemic, and to the best of our knowledge, those that have been conducted have employed linear methodologies. Therefore, this paper aims to assess the extent to which the presence of a chronic physical illness, one’s COVID-19 threat perception, positive dyadic coping, and the quality of the couple’s relationship have an impact on anxiety, depression and stress in individuals living in an intimate relationship in Portugal under COVID-19 confinement. In addition, the use of linear and nonlinear methodologies enables this paper to produce complementary results that can improve the understanding of the problem.

In line with our objective, the findings were consistent with our expectations: linear analyses showed that high COVID-19 threat perception and low couple relationship quality were significantly associated with symptoms of anxiety, depression, and stress. These results are in line with other studies that pointed out the relevance of illness representation for a person’s emotional adjustment [14, 34]. They also show that individuals with a better-quality couple’s relationship enjoy more protection in the face of adversity [35, 36], considering that this variable was among the most relevant in relation to lower levels of anxiety, depression, and stress in both linear and non-linear models in our study, these findings align with previous cross-cultural research highlighting the importance of relationship-related factors in emotional adjustment during adverse situations [16].

However, consistent with some studies, it is striking that the presence of a chronic physical illness is not a relevant variable for the appearance of anxiety, depression or stress during the pandemic [15, 37]. This result has also been found with samples from Spain, but it needs to be explored further in other contexts in the future [14, 15]. In fact, other studies had already pointed out that this factor could have a great impact on people during the pandemic by showing that the pandemic negatively affected almost half of those with a physical health problem by changing their exercise routines or the frequency of medical check-ups and testing, and by decreasing access to medical monitoring and treatment [38]. These aspects may have led case-control studies to find that people with a physical health problem had higher levels of distress and somatization than people without physical health problems [39], meaning that facing two potential stressors may increase the chances of suffering distress. However, and in a converse sense, it may well be that individuals who live with a chronic physical illness on a daily basis may have already acquired their own personal resources and learned strategies to cope with any situation of adversity that may arise from a health crisis [40, 41].

A notable finding appears in the fact that the linear methodologies do not designate positive dyadic coping as being a variable of interest for coping with adversity in this sample. This result is striking since the literature [29] had pointed out that dyadic coping is highly relevant for handling stress when individual resources are exhausted. Moreover, positive dyadic coping proved to be a protective variable in a cross-cultural study conducted during the pandemic [16].

Nonetheless, in our study nonlinear models seem to shed new light on the understanding of the situation. For example, the different configurational pathways linked to high levels of anxiety symptoms commonly involved the presence of a chronic physical illness and a high perceived threat of illness, combined with the absence or presence of dyadic coping or relationship quality. In these cases, we perceive that although there is positive dyadic coping and a high perceived quality of the couple’s relationship, the presence of a high perceived threat of the pandemic and a chronic physical illness generates anxiety. Likewise, regarding the low levels of symptoms of anxiety, we note that the combinations found point to the importance of not having a previous chronic physical illness, of having a low perception of threat from the illness, and of having high positive dyadic coping or high relationship quality. Thus, we observe how in the case of anxiety, the presence of a high COVID-19 threat perception is very relevant in combination with the presence of a chronic physical illness and the absence of protective relationship factors from the partner.

In the explanation of low levels of depression, the interaction between the absence of a chronic physical illness, with the absence of COVID-19 threat perception, and the presence of positive dyadic coping and relationship quality, proved to be very important. In this sense, it seems that both non-modifiable factors (such as not having a previous physical illness) and modifiable factors for both the individual and relationship may influence the absence of symptoms of depression. Previous work has pointed to the possible psychological impact of chronic physical illness in the face of adversity [13]. As previously mentioned, it might therefore be expected that coping with two potential stressors, the pandemic and chronic physical illness, would influence psychological adjustment [42]. However, not having a chronic illness, as a single factor, does not associate to low levels of depression; this particular outcome points to the combination of that condition with a non-threatening perception of COVID-19 and the presence of positive dyadic coping and good relationship quality. Therefore, we can see how variables related to the partner are relevant for mental health, as other studies have advanced [35, 36, 42]. Furthermore, even in cases where an individual has a poor-quality relationship, when he/she presents high positive dyadic coping, high levels of depression may not be present. In these cases, our data show that it is possible that the presence of adequate coping skills for a person living with a partner is more relevant than the quality of the couple’s relationship.

Still, as for the explanation of stress through nonlinear models, the different combinations indicated the relevance of COVID-19 threat perception as well as the presence of high levels of relationship quality and positive dyadic coping. The first two variables appear particularly relevant, as certain configurations indicated that high levels of stress were still observed when high COVID-19 threat perception co-occurred with low relationship quality, even in the presence of high positive dyadic coping.

From the comparison of the two methodologies, perceived COVID-19 threat, and relationship quality emerged as the most consistently related factors to symptoms of anxiety, depression and stress in individuals living with a partner during the pandemic in Portugal, which falls in line with research carried out separately in other countries [16, 43, 44]. In this sense, it can be observed how one’s appraisal and beliefs about the illness can relate to psychological distress: the person’s perception of the disease is the most relevant to his/her adjustment, as it determines his/her behavior [45]; the same way as a high quality of the couple’s relationship can buffer the effects of stressful life events [46].

About the importance of having a chronic physical illness and its impact on psychological distress during the COVID-19 confinement, we can state that in the QCA models, as opposed to linear methodology, it is evident that the presence of this condition, along with the absence of positive dyadic coping, played an important role in psychological adjustment to this adversity. This is possible because QCA models often provide a more complete picture of reality [47]. Therefore, through the different combinations, we can gain a deeper and more comprehensive understanding of reality.

Despite the contributions, this study has some limitations that should be considered. First, the cross-sectional design and use of an online convenience sample—mainly composed of women—limit the generalizability of the results. In particular, the high proportion of female participants (83.9%) may have influenced the findings, potentially limiting their applicability to male populations or more gender-balanced contexts. Future research should aim to include more balanced samples or implement stratified analyses to examine possible gender differences and improve the external validity of the results. Nonetheless, this gender imbalance reflects common patterns in psychological research participation.

Second, data collection occurred during a highly specific historical period (COVID-19 confinement), which, while contextually relevant, also introduces potential confounders that limit longitudinal applicability. Finally, the internal consistency of the B-IPQ in our sample was low (Cronbach’s α = 0.50), which may affect the reliability of the results. This limitation suggests that the items may not have captured the construct of illness perception in a consistent manner, potentially introducing measurement error. As a result, the associations found between illness perception and psychological distress should be interpreted with caution, as the low reliability may have attenuated or obscured potential relationships. Future studies should consider using alternative or adapted versions of the B-IPQ with better psychometric properties in similar populations. If future studies are conducted cross-culturally, more generalizable results can be found. In addition, protocols for early psychological assessment and intervention in situations of crisis may also be developed. Moreover, research should be conducted considering the dyad as a whole and compare the results of all actors.

In conclusion, it is essential to consider the importance of relationship variables related to the well-being of persons living with a partner as factors that offer protection in the face of adversity, particularly those highlighting the quality of the couple’s relationship and positive dyadic coping. But attention must also be paid to individual variables such as the perceived threat of the disease.

The present findings are encouraging, as they suggest that the positive relationship-oriented coping resources of individuals living in an intimate relationship can enhance a person’s psychological adjustment to stress.

This research highlights the importance of addressing both individual perceptions of health threats and the quality of interpersonal relationships to mitigate the psychological impact of pandemics on individuals living with partners. The findings indicate that psychological interventions and public health strategies should go beyond managing the direct consequences of a health crisis to also support relational dynamics and coping mechanisms within intimate partnerships.

Strengthening relationship quality and promoting positive dyadic coping may be essential components of effective mental health strategies during large-scale health emergencies. These interventions can be delivered through community health services, online platforms, or through targeted campaigns that aim to enhance couples’ resilience facing shared stressors.

Moreover, the study’s insights into the relative resilience of individuals with chronic illnesses suggest the value to integrate personalized coping strategies into mental health support frameworks. Healthcare providers and policymakers should consider tailored support programs that build on the existing adaptive strategies of those with chronic conditions while addressing the specific challenges posed by crises like the COVID-19 pandemic. Promoting relational coping and recognizing the diverse effects of stress across population groups may help protect mental health more broadly during times of crisis.

Furthermore, these fsQCA findings underscore the relevance of a configurational approach to psychological distress, as they reveal that similar levels of anxiety, depression, or stress may arise from distinct combinations of individual vulnerabilities and relational dynamics. Theoretically, this aligns with systemic and transactional models of stress, suggesting that psychological outcomes are not determined by isolated factors, but by the interplay of multiple conditions acting jointly. Practically, this perspective encourages the development of personalized intervention strategies that target specific risk profiles rather than isolated variables. For example, individuals with high perceived threat and chronic illness may benefit more from targeted interventions aimed at enhancing relationship quality and dyadic coping, while those lacking a supportive partnership may need individualized coping resources. Thus, the configurational insights from fsQCA offer a valuable framework for tailoring psychosocial support strategies during collective crises like pandemics.

## Conclusions

This study revealed that during the COVID-19 pandemic in Portugal, individuals in intimate relationships experienced significant psychological distress when they perceived a high threat of COVID-19. In contrast, strong relationship dynamics, such as high-quality couple relationships and effective dyadic coping, provided meaningful protection against anxiety, depression, and stress.

Interestingly, the presence of a chronic physical illness only was not significantly associated with increased distress, possibly reflecting the use of resilient coping strategies among individuals living with such conditions.

These findings emphasize the importance of focusing on both individual perceptions of health threats and the quality of interpersonal relationships to mitigate the psychological impacts of pandemics.

## Availability of Data and Materials

The datasets generated and/or analyzed during the current study are available from the corresponding author upon reasonable request.
